# Melting enhancement of PCM in a finned tube latent heat thermal energy storage

**DOI:** 10.1038/s41598-022-15797-0

**Published:** 2022-07-07

**Authors:** Sameh Ahmed, Aissa Abderrahmane, Abdulkafi Mohammed Saeed, Kamel Guedri, Abed Mourad, Obai Younis, Thongchai Botmart, Nehad Ali Shah

**Affiliations:** 1 College of Science, King Khalid University, Abha, 61413 Saudi Arabia; 2 Department of Mathematics, Faculty of Science, South Valley University, Qena, 83523 Egypt; 3 Laboratoire de Physique Quantique de La Matière et Modélisation Mathématique (LPQ3M), University of Mascara, Algeria, Algeria; 4 Department of Mathematics, College of Science, Qassim University, Buraydah, Kingdom of Saudi Arabia; 5 Mechanical Engineering Department, College of Engineering and Islamic Architecture, Umm Al-Qura University, P.O. Box 5555, Makkah, 21955 Saudi Arabia; 6 College of Engineering at Wadi Addwaser, Mechanical Engineering Department, Prince Sattam Bin Abdulaziz University, WadiAddwaser, Saudi Arabia; 7 Department of Mathematics, Faculty of Science, Khon Kaen University, Khon Kaen, 40002 Thailand; 8 Department of Mechanical Engineering, Sejong University, Seoul, 05006 South Korea

**Keywords:** Energy infrastructure, Mechanical engineering

## Abstract

The current paper discusses the numerical simulation results of the NePCM melting process inside an annulus thermal storage system. The TES system consists of a wavy shell wall and a cylindrical tube equipped with three fins. The enthalpy-porosity method was utilized to address the transient behavior of the melting process, while the Galerkin FE technique was used to solve the system governing equations. The results were displayed for different inner tube positions (right-left–up and down), inner cylinder rotation angle (0 ≤ α ≤ 3π/2), and the nano-additives concentration (0 ≤ ϕ ≤ 0.04). The findings indicated that high values of nano-additives concentration (0.4), bigger values of tube rotation angle (3π/2), and location of the tube at the lower position accelerated the NePCM melting process.

## Introduction

Energy storage is critical in thermal systems that use intermittent energy sources such as solar energy. Although less difficult, sensible heat storage needs large volumes to store the storage material and also exhibits temperature change throughout the charge/discharge cycles^1,2^. On the other hand, latent heat thermal energy storage (LHTES) systems have a large thermal heat capacity, high energy storage density, negligible temperature change throughout the charge /discharge cycles, wide phase transition temperature range, and low cost^3,4^. These advantages enable them to have a wide range of applications in solar energy utilization^5,6^, energy-efficient buildings^7^ and domestic hot water^8^, load management^9^, refrigeration and air conditioning^10^, and industrial waste heat recovery^11,12^. As a result, incorporating PCM-based TES in a variety of thermal applications is a hot topic of research. Narasimhan et al.^13^ conducted a thermal examination of a storage unit with several PCM with high conductivity particles dispersed. The findings indicate that the performance of the 3-PCM unit is improved when the second and third PCMs are positioned with latent heats greater than the first PCM. Abdelgaied et al.^14^ investigated a combination of various successful modifications to the design of a pyramid solar distiller (PSD) to maximize its cumulative output. One of these changes is the addition of PCM with pin fins. The findings indicate that this new combination of modifications uses this new combination of modifications Khdair et al.^15^ investigated ways to reduce energy consumption in buildings by using PCM-23 and taking into consideration Riyadh's yearly average temperature. They discovered that although the use of PCM-23 was ineffective during the warm months, it reduced heat exchange during the cooler months, resulting in a 3984-kWh reduction in energy usage over the course of a year. Kurnia et al.^16^ carried out an experimental study to determine the energy storage effectiveness of a Hybrid TES with a PCM layer serving as both an insulation and an energy storage layer. The experimental results indicate that Hybrid TES with PCM wall layer provides better heat insulation than traditional sensible TES, as demonstrated by a higher withheld temperature within the storage medium. Shamsi et al.^17^ mathematical modeling to evaluate and optimize the encapsulated cascade PCM latent TES performance. Their investigation examined the effects of various parameters such as the location, type, and amount of PCM. They determined that the cascade PCM thermal storage system outperforms the single PCM thermal storage system. Sharaf et al.^18^ evaluated a passive cooling technology that combines an aluminum metal foam (AMF) and PCM to regulate the temperature of a photovoltaic (PV) system (PV-PCM/AFM). The results indicated that the power produced by the PV-PCM/AFM system was 1.85 percent, 3.38 percent, and 4.14 percent higher than that of conventional PV during December, January, and February respectively.

Heat energy storage systems offer the benefits of high energy storage efficiency and consistent temperature due to the use of phase change material (PCM); however, its disadvantage is that thermal energy storage takes longer to complete due to the material poor thermal conductivity. New technologies are being developed to address this inadequacy, including ones that increase heat transfer^19,20^. Several researchers develop highly conductive PCMs composite by dispersing nanoparticles in conventional PCM to promote the heat transfer rate of PCMs^21–23^. He et al.^24^ studied the synthesis and thermal characterization of several composite NEPCMs based on graphene nanosheets (GNPs), multi-walled carbon nanotubes (MWCNTs), and nano graphite (NG). Their findings indicated a considerable increase in thermal conductivity as a consequence of the addition of nanoparticles (particularly GNPs) to myristic acid PCM. Chu et al.^25^ sought to enhance the ventilating unit efficiency by utilizing an RT28 PCM. CuO nanoparticles were distributed in RT28 to improve their heat absorption. Soliman et al.^26^ developed an effective waste heat recovery solution for diesel engines utilizing NEPCM. Theoretically, Ghalambaz et al.^27^ explored the non-Newtonian phase-transition of NEPCM with mesoporous silica particles in an inclined container using a deformed mesh method. Al-Waeli et al.^28^ enhanced the efficacy of the PCM/ PVT system by including SiC nanoparticles in PCM. Electrical and thermal efficiency levels of roughly 14 percent and 72 percent, respectively, were achieved in the experiments. Kazemian et al.^29^ enhanced the thermal efficiency of PCM by using Al2O3 nanoparticles. They introduced NEPCM to a PVT-water collection unit and examined the resulting system experimentally. Fan et al.^30^ studied the impact of different carbon nanofillers on the thermal conductivities of PCMs in an experimental setting. The findings indicate that with a 5.0 wt percent load, the maximum thermal conductivity of nano-PCMs was increased nearly 1.7 times, which aided in enhancing PCMs' heat transfer efficiency. Nada et al.^31^ Experimental testing investigated the efficacy of employing Al2O3 nanoparticles dispersed in PCM as a heat dissipation system for cooling photovoltaic modules. The electrical performance of the suggested system was increased by 6.8 percent and 12.1 percent, respectively, when Al2O3 nanoparticles were dispersed within the PCM. Singh et al.^32^ studied the thermal enhancement properties of a binary eutectic PCM containing varying amounts of graphene nanoplatelets. The total melting time was reduced by 17.3 percent when graphene nanoplatelets were used at a concentration of 5% in comparison to the pristine PCM heat exchanger.

Recently, numerous external approaches for improving heat transmission in PCM have been investigated, including the use of wavy surfaces to increase the contact surface toward greater heat exchange rates and the use of inner spinning cylinders to promote convective flow inside the melted PCM. Kashani et al.^33^ investigated the solidification of a copper–water nanofluid in a two-dimensional hollow with vertically wavy walls. They demonstrated that the solidification period could be regulated by varying the surface waviness, which improves the domain heat transmission performance. Abdollahzadeh et al.^34^ investigated the solidification of Cu-water nanofluid in a vertical enclosure with various wavy surfaces, including divergent-convergent and convergent-divergent walls. They demonstrated the increased heat transmission rate by employing sinusoidal wavy walls. shahsavar et al.^35^ examined the performance of a vertical double-pipe LHTES system with sinusoidal wavy channels. The results indicated that the average heat transmission rate for the wavy channel composite PCM case is 10.4 and 18.9 times that for the smooth channel pure PCM case during the melting and solidification processes, respectively. Alizadeh et al.^36^ established a numerical approach for modeling and optimizing the solidification process in a Latent Heat TES System composed of a wavy shell and a fin-assisted tube. Rotating cylinders may be utilized to control convection inside a cavity in a variety of heat transfer applications. Numerous research on the influence of revolving cylinders on the phase transition of PCMs have been reported recently^37–40^. Selimefendigil et al.^41^ explored mixed convection in a square cavity filled with PCM under the influence of a spinning cylinder. It was discovered that the parameters of the revolving cylinder may be utilized to regulate the heat transport and melting processes inside the cavity. Al-Kouz et al.^42^ conducted a comprehensive numerical investigation of entropy generation and mixed convection in a three-dimensional cavity filled with a phase change material (PCM) and including a revolving cylinder. The findings suggested that impeding the cylinder with an angular velocity increased the heat transfer rate by 21.2% compared to a static cylinder. Sadr et al.^43^ investigated mixed convection of water-NEPCM inside a square enclosure with cold boundaries and a revolving hot cylinder at the core. According to their findings, increasing the Re and Gr values increased heat transmission rate. However, when the Gr number is large, increasing the rotational velocity of the inner cylinder and hence the Re number reduces Nusselt. Additionally, the data indicate that supplementing with NEPCM may enhance the Nusselt number by more than 13%.

Based on the aforementioned literary survey, there are few studies concerning with the melting enhancement of PCM in wavy finned tubes latent heat thermal energy storage are presented. One of these studies is that of Elmaazouzi et al.^44^ where the enhancement of the thermal performance of finned latent heat thermal energy storage system was examining. Therefore, the main objective of this investigation is to examine the impacts of the non-regular outer boundaries together with heated fins attached with a rotating cylinder on the heat transfer rate, melting rate and temperature distributions. Also, the 2nd law of the thermodynamic is applied to analyse the system entropy and values of the Bejan number. Furthermore, the thermal energy storage systems are considered a direct practical application of this purpose.

## Problem description

**Figure 1:**
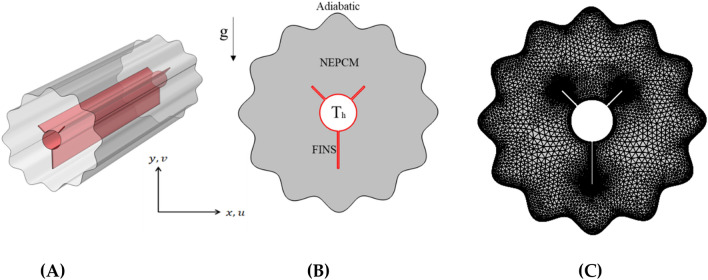
(**A**) 3D model of the shell and tube TES with embedded fins (**B**) A two-dimensional illustration of the studied model with boundary conditions. (**C**) A mesh sample.

**Figure 2:**
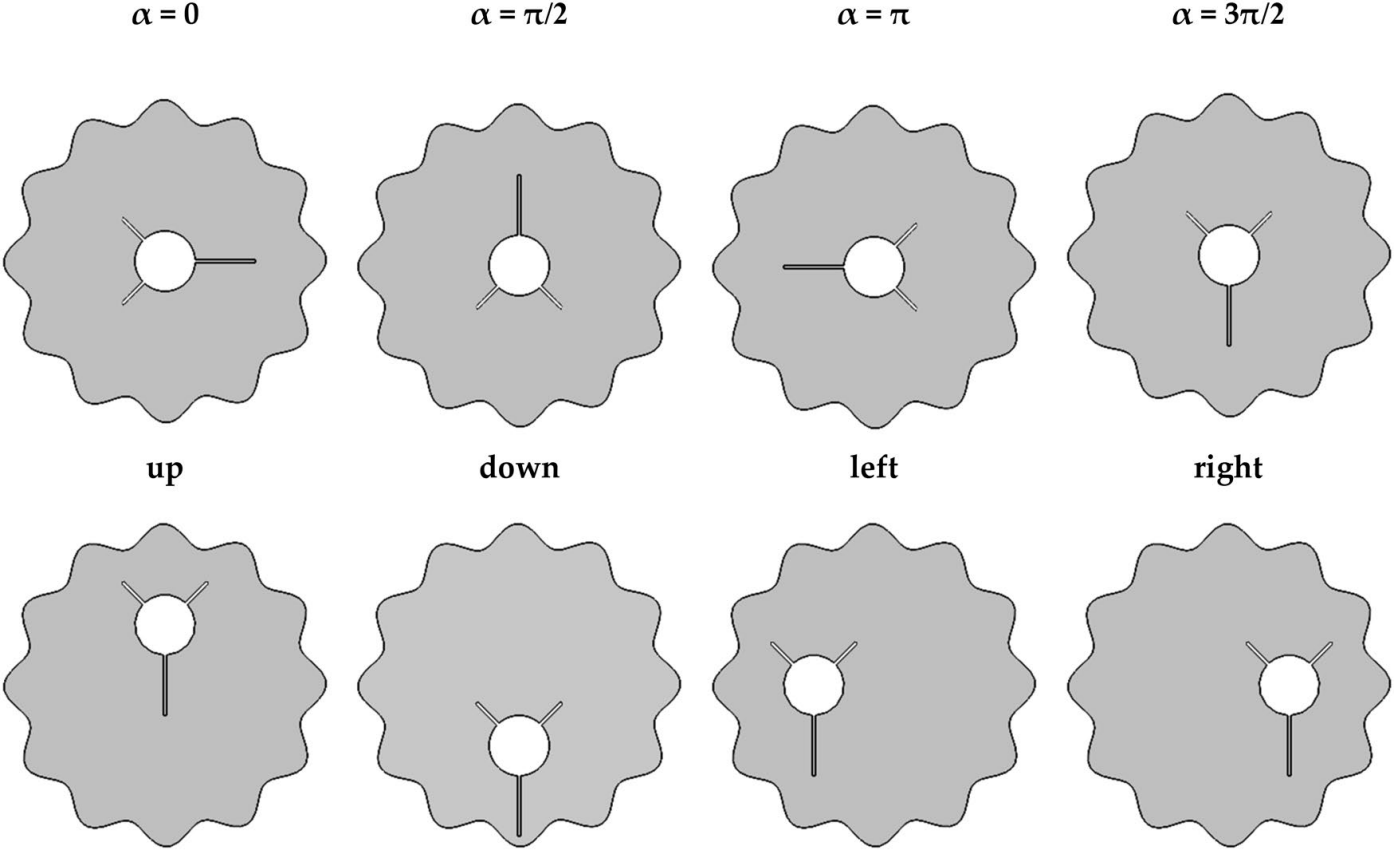
Different cases considered for orientation and positioning of the finned tube in this study.

Optimizing the charging and discharging of a PCM inside a shell-and-tube heat exchanger operating as a TES device requires investigating complicated thermofluid processes, which are addressed in this work using computational fluid dynamics (CFD) methods. The three-dimensional configuration investigated in this article is depicted in Fig. 1A. It is a horizontally oriented eccentric annulus contained inside a phase-change material-filled outer cylinder. To save CPU time, the length of the TES is set to be long enough to simplify the issue to the two-dimensional configuration presented in Fig. 1 with boundary constraints (B). The suggested enclosure is a two-dimensional concentric annulus with a wavy tube on the outside and a finned circular tube on the inside. The outer shell is adiabatic, while the inner finned tube maintains a constant temperature Th. Inner and outer tubes (for circular cylinders) have 2 cm and 8 cm diameters, respectively. Three tiny fins with a thickness of 0.1 cm are affixed to the inner tube (two small ones are 2 mm in length and one large one is 3 mm in length). This research investigates several fin orientations (α = 0, π/2, π and 3π/2) and inner tube locations (see Fig. 2). The enclosure net area (PCM zone) is identical in all cases.

Paraffin wax is used as the phase change medium, while copper nanoparticles are used to increase the PCM's thermal conductivity. The thermophysical properties of paraffin wax and copper nanoparticles are summarised in Table 1.

**Table 1:** Lists the characteristics of both nanoparticles and the PCM.

Property	PCM (liquid)	PCM (solid)	Nanoparticles
Density, ρ (kg/m^3^)	775	833.6	8954
Specific heat Cp (kJ/kg K)	2.44	2.384	0.383
Thermal conductivity, K (W/m K)	0.15	0.15	400
Melting temperature, T_m_ (K)	54.32		–
Kinematic viscosity, μ (m^2^/s)	8.31 × 10^–5^		–
Latent heat of fusion, H (kJ/kg)	184.48		–
Thermal expansion coefficient (K^−1^)	7.14 × 10^–3^		1.67 × 10^−5^

### Mathematical model

The enthalpy-porosity methodology is the most frequently used technique for analyzing unstable heat transport problems, such as PCM phase transitions. The enthalpy-porosity technique is utilized to simulate the melting and heat transmission characteristics of embedded fins in NePCM. The advantage of the enthalpy-porosity technique is that it does not require direct monitoring of the phase interface; rather, it generates the energy equation throughout the whole calculation domain using enthalpy and temperature. Due to the substantial nonlinearity of the phase change process, its issues become more intricate. The following parameters are considered to streamline the computation:

1. The flow of liquid NePCM is regarded to be incompressible and laminar.
2. Natural convection is disregarded for the minuscule density shift in PCM caused by gravity and density difference during phase transition.
3. Since the physical properties of PCM remain relatively consistent during the phase transformation, they may be deemed constant.
4. Overlooking the volumetric impact of viscous dissipation and heat source.
5. The container wall is assumed to maintain a constant temperature, ignoring the heat transfer resistance of the container wall and the convective heat transfer process inside the tube.
6. There is no heat transfer Between the shell and its surroundings

Based on the above assumptions, it is possible to develop the governing equation for the melting process of NePCM in the wavy finned enclosure. The equations may be expressed as follows:

**Continuity equation:**

1 $$\nabla \cdot (\overrightarrow{V})=0$$

**Momentum equation:**

2 $$\frac{\partial ({\rho }_{np}\overrightarrow{V})}{\partial t}+\nabla \cdot ({\rho }_{np}\overrightarrow{V})=-\nabla P+{\mu }_{np}{\nabla }^{2}\overrightarrow{V}-{S}_{b}+{S}_{a}$$

where the subscriptions np refers to the nano-enhanced PCM and ${S}_{a}$ a is the source term for the porosity function proposed by Bernt et al.^45^

3 $$ S_{a} \,\,\,{\text{define}}\,\,{\text{as}}\,\,S_{a} = - A\vec{V}\,\,\,{\text{with}}\,\,\,A = \frac{{(1 - \eta )^{2} }}{{\left( {\pi^{3} + 10^{ - 3} } \right)}}C $$

4 $$ {\text{And}}\,\,\nabla \left( P \right) = - \frac{{(1 - \eta )^{2} }}{{\eta^{3} }}C \cdot \vec{V} $$

${S}_{b}$ is the Boussinesq approximation to the buoyancy force; the value is as follows:

5 $${S}_{b}={\left({\rho }_{ }\beta \right)}_{np}\left(T-{T}_{h}\right)\overrightarrow{g}$$

The vector of fluid velocity is denoted by $\overrightarrow{V}$. The two-dimensional model specifies the axial and radial velocity vector components as follows:

6 $${V}_{axial}=v{ and V}_{radial}=u$$

7 $$ {\text{Energy equation}} \,\,\frac{\partial \left( H \right)}{{\partial t}} + \nabla \cdot \left( {\vec{V}H} \right) = \nabla \cdot \left( {k_{np} \nabla T} \right) $$

where H signifies a certain enthalpy and is stated in the following manner:

8 $$H=h+\Delta H$$

where h is a sensible enthalpy denoted by the formula:

9 $$h={h}_{ref}+{\int }_{{T}_{ref}}^{T}{\left(\rho {c}_{p}\right)}_{np}dT$$

ΔH: latent heat changing step during the phase of the PCM changed between solid to liquid, the value of $C$ is taken as $C={10}^{6}$.

Additionally, $\eta$ is the equation for the liquid portion of the liquid/solid zone, which assists in defining the zone of calculated cells, where the liquid zone equals $\eta =1$ and the solid zone equals $\eta =0$ , while the mushy zone equals $0<\eta <1$, and can be expressed as follows :

10 $$\eta (T)=\left\{0 if T<{T}_{s} 1 if T>{T}_{l} \frac{T-{T}_{s}}{{T}_{l}-{T}_{s}} if {T}_{l}>T\ge {T}_{s}\right\}$$

with ${T}_{l}$ and ${T}_{s}$ denoting the NePCM's liquid and solid temperatures, respectively.

It is possible to represent the liquid fraction expression as flows:

11 $$\eta (T)=\left\{0\,\, if\,\, T<\left({T}_{m}-\Delta T\right) 1 if T>\left({T}_{m}+\Delta T\right) \frac{\left(T-{T}_{m}+\Delta T\right)}{2\Delta T} if \left({T}_{m}+\Delta T\right)>T\ge \left({T}_{m}-\Delta T\right)\right\}$$

The thermophysical characteristics of paraffin wax are employed in the situations of pure PCM, and in the cases of NePCM, the parameters are estimated using a mix of paraffin wax and copper nanoparticle properties, as shown in Table 1. The preceding equations use general notations for the thermophysical characteristics and are applicable to both pure PCM and NePCM. The density and specific heat capacity of the nano-PCM material are estimated as follows:

12 $$ \rho_{np} = \left( {1 - \varphi } \right)\rho_{p} + \varphi \rho_{n} $$

13 $$ \left( {\rho c_{p} } \right)_{np} = \left( {1 - \varphi } \right)\left( {\rho c_{p} } \right)_{p} + \varphi \left( {\rho c_{p} } \right)_{n} $$

where the subscriptions n and p refer to the nanoparticles and PCM, respectively.

Here, $\varphi$ represents the volume fraction of nanoparticles added in the PCM.

Similarly, the latent heat of fusion, the effective thermal conductivity, and the thermal expansion coefficient of NEPCM can be found using the following set of equations^46^.

14 $$ \left( {\rho L} \right)_{np} = \left( {1 - \varphi } \right)\left( {\rho L} \right)_{p} $$

15 $$ k_{np} = \frac{{k_{n} + 2k_{p} - 2\varphi \left( {k_{p} - k_{n} } \right)}}{{k_{n} + 2k_{p} + \varphi \left( {k_{p} - k_{n} } \right)}}k_{p} $$

16 $$ (\rho \beta )_{np} = \left( {1 - \varphi } \right)(\rho \beta )_{p} + \varphi (\rho \beta )_{n} $$

The entropy created as a result of thermal irreversibility (heat transfer) equals

17 $$ S_{ht} = \frac{{k_{nf} }}{{\overline{T}^{2} }}\left[ {\left( {\frac{{\partial \overline{T} }}{\partial x}} \right)^{2} + \left( {\frac{{\partial \overline{T} }}{\partial y}} \right)^{2} } \right] $$

The entropy created as a result of the flow's irreversibility (presence of a friction factor) is equal to

18 $$ S_{f} = \frac{{\mu_{nf} }}{{\overline{T} }}\left\{ {2\left[ {\left( {\frac{{\partial \overline{u} }}{\partial x}} \right)^{2} + \left( {\frac{{\partial \overline{v} }}{\partial y}} \right)^{2} } \right] + \left( {\frac{{\partial \overline{u} }}{\partial x} + \frac{{\partial \overline{v} }}{\partial y}} \right)^{2} } \right\} $$

The total entropy, which comprises the entropy increase caused by heat transfer and fluid friction, and the Bejan number, which is the ratio of irreversible heat transfer to total entropy, are computed using the following formulae.

19 $$ S_{tot} = S_{ht} + S_{f} $$

20 $$ Be = \frac{{S_{ht} }}{{S_{tot} }} $$

### Validation and mesh independence study

**Figure 3:**
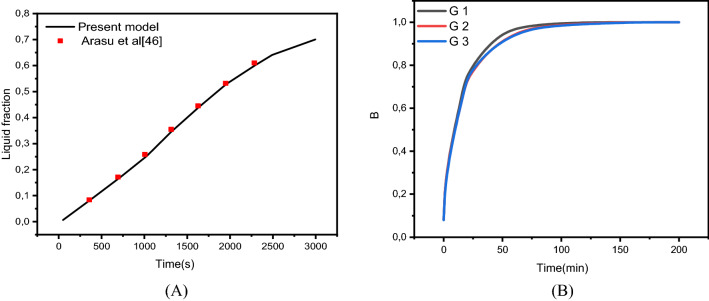
(**A**) Comparison of numerical results with^46^. (**B**) Grid independent study.

To verify the implementation of mathematical modeling of melting and the solution methodology outlined above, the result obtained for melting interface propagation in a square enclosure was compared to the simulation solution provided in Arasu et al.^47^ in Fig. 3A. The present findings are highly consistent with those previously published, implying that the numerical model's fundamental validity has been established.

The mesh independence study is established by examining the average liquid fraction over time for the various grid sizes shown in Table 2. Figure 1C shows an example of the computing grid, where the mesh must be finely tuned throughout the domain to adjust to the moving of the melting interface at each time step. The influence of multiple mesh sizes on the liquid fraction during the melting process is shown in Fig. 3B. The mesh G2 is selected to conduct all numerical simulations in this research based on the outcomes of the mesh independence study (illustrated in Fig. 3B).

**Table 2:** Numerical test results for grid independency study.

Mesh	G1	G2	G3
Number of elements	25,096	66,982	100,384

## Results and discussion

The major outcomes are represented in terms of the features of velocities, temperature fluid fraction irreversibility, Bejan number and local liquid fraction. The average values of the temperature T_avg_, Nusselt number Nuavg, melting rate β and Bejan number are shown in 2D illustrations. It is interesting, here, in the examination of the rotation impacts (0 ≤ α ≤ 3π/2), the position of the rotating shape (right -up-down) and concentration of the NP (nanoparticles) (0 ≤ ϕ ≤ 0.04). Furthermore, the transient case of PCM flow is focused with a wide range of time: 0 < Time ≤ 1600 s.

**Figure 4:**
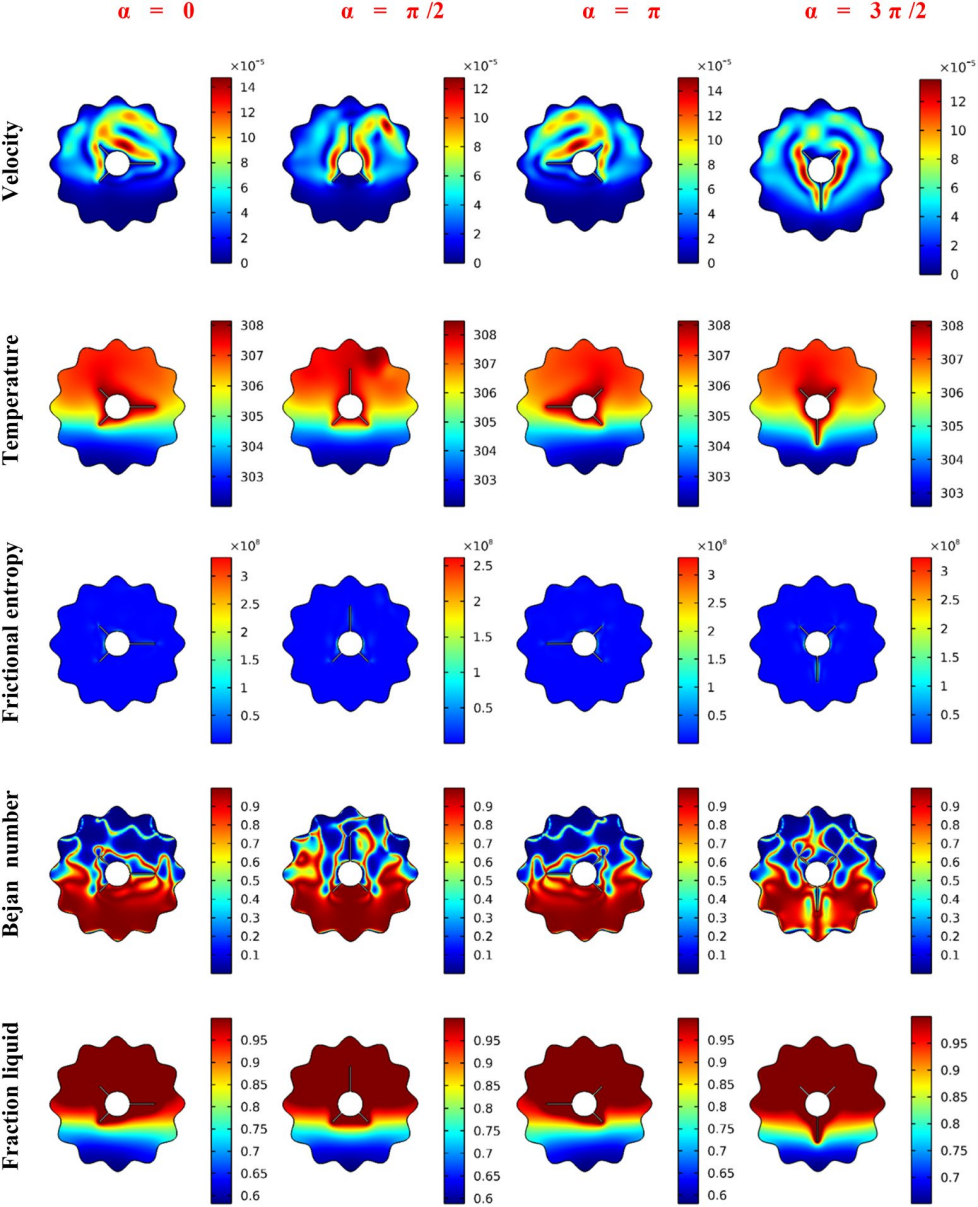
The impact of the finned tube orientation on the Velocity, Temperature, Frictional entropy, Bejan number, and Liquid fraction contour after 30 min.

Figure 4 depicts the floods of velocities, isotherms, viscous dissipation entropy, local Be number and melting process for various values of the rotation angle α. Here, it should be mentioned that the inner rotating shapes are three heated fins attached to a circular cylinder. The flow is concentrated in the upper half of the wavy domain in the cases of α = 0, π/2 and π, while in the case of α = 3π/2, the flow is seen in the whole domain. The benchmark values of the velocities are higher in cases of α = 0, π than those of α = π/2, 3π/2. The temperature floods show heated zones around the inner rotating shapes and near the upper wavy boundaries, while the cold features occur on the lower edge. The rotation of the inner shape causes a redistribution of the heated/cold areas within the wavy domain. Also, the thickness of the thermal edge layers is noted to be higher in case α = 3π/2 compared to the other cases. In the same context, due to the higher velocity gradients around the rotating shape, the fluid friction entropy occurs in these regions for all values of π/2.

Additionally, features of the local Bejan number illustrate that the heat transfer irreversibility in the lower area is more dominant than the irreversibility owing to the fluid fraction. Also, the case of α = 3π/2 decreases the HT irreversible process. The variations of inner-shape rotation also influence the melted process. It is gathered in the upper area, and a redistribution of the melting area is obtained as α is altered.

**Figure 5:**
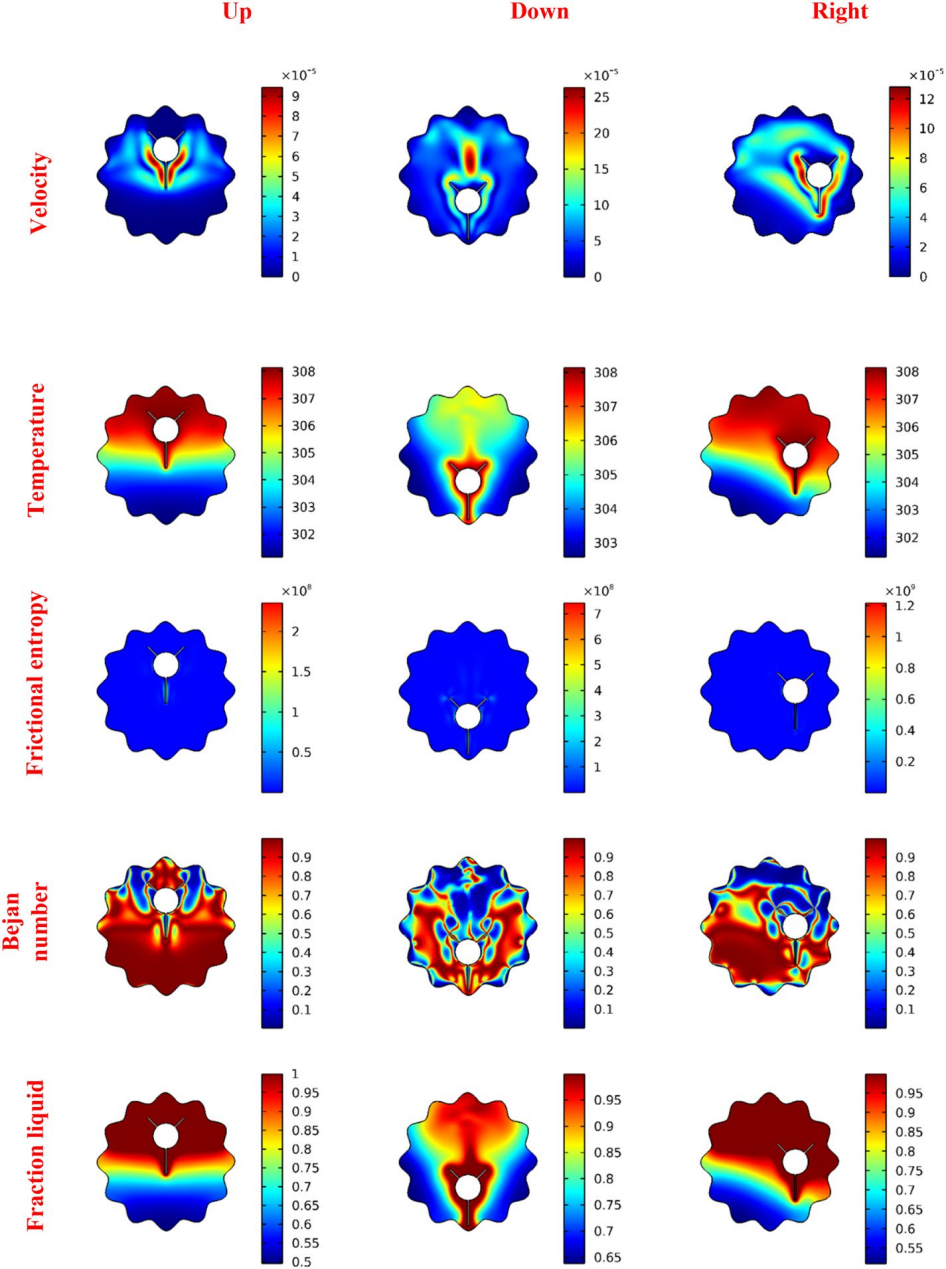
The impact of the finned tube position on the velocity, temperature, frictional entropy, Bejan number, and Liquid fraction contour after 30 min.

In Fig. 5, the floods for velocities, temperature, fluid friction irreversibility, local Be number and melting process for various locations of the inner rotating shapes are illustrated. Here, four locations of the rotating cylinder-fins are considered, namely, Up, Down, Left and Right. Different configurations for the flow and thermal fields are taken as the location changes. The results revealed that when the rotating fins are placed in the upper part (Up-case) of the wavy domain, this causes the smallest flow rate, and the HT rate, as well as the heated zone, is decreased. On the contrary, the flow is accelerated, and the thickness of the thermal boundary layer is enhanced when the rotating heated fins are placed in the bottom of the wavy domain (down case). Additionally, features of the Be number show that the HT irreversibility is overriding on most of the wavy area than the viscous dissipation irreversibility in the Up-case while the melting process is reduced in this case. The fluid friction entropy has higher values in the down-case, and a strong melting process is obtained.

**Figure 6:**
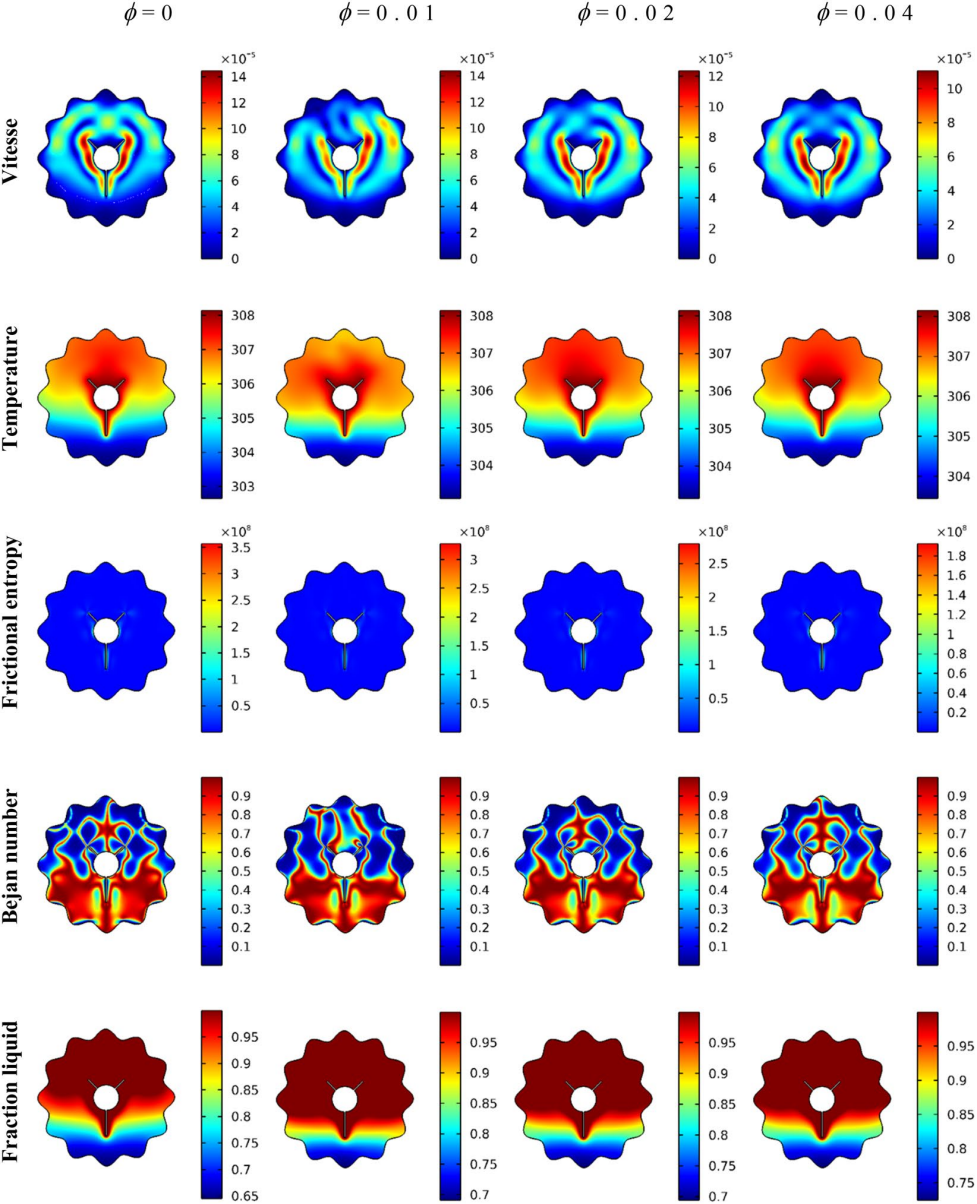
The impact of the NP concentration on the velocity, temperature, frictional entropy, Bejan number, and Liquid fraction contour after 30 min.

The flow features, thermal fields, viscous dissipation irreversibility, local Bejan coefficient and rate of the melting within the wavy domain with variations of the NP concentration 0% ≤ ϕ ≤ 4% are displayed in floods mode as depicted in Fig. 6. Remarkably, stretching of the inner eddies, the strength of the mixture flow and isotherms distributions are diminishing as ϕ is rising. Physically, these behaviors are due to the dynamic suspension viscosity enhanced as ϕ is growing, and the flow rate is reduced. Also, the velocity gradients are weak as ϕ is growing, resulting in a clear reduction in the fluid friction entropy. Thus, features of Be number show a clear dominance of the HT irreversibility as ϕ is increased. Further, the expected behavior is noted in this Figure. That is the increase in the melting situation as NP concentration is enhanced.

**Figure 7:**
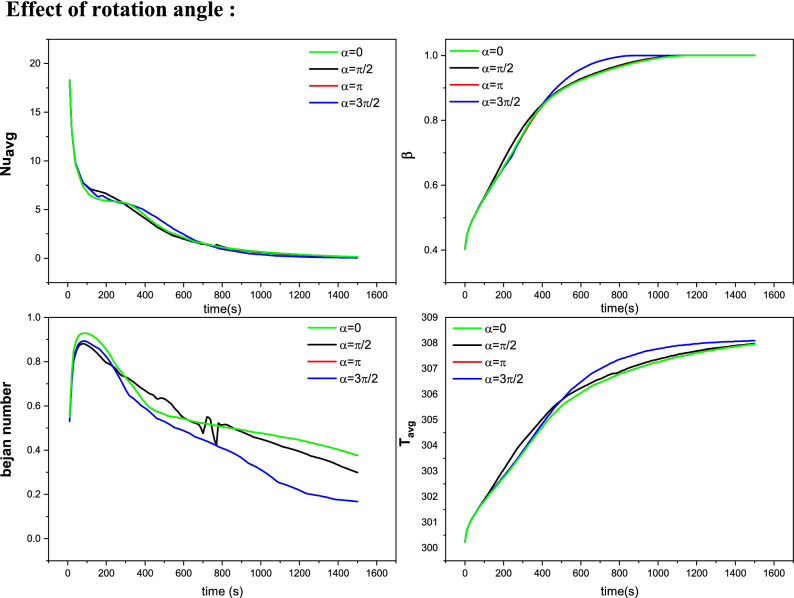
The influence of the rotation angle (α) on the Average temperature, Average Nusselt number, Average liquid fraction (β) and Bejan number over time.

**Figure 8:**
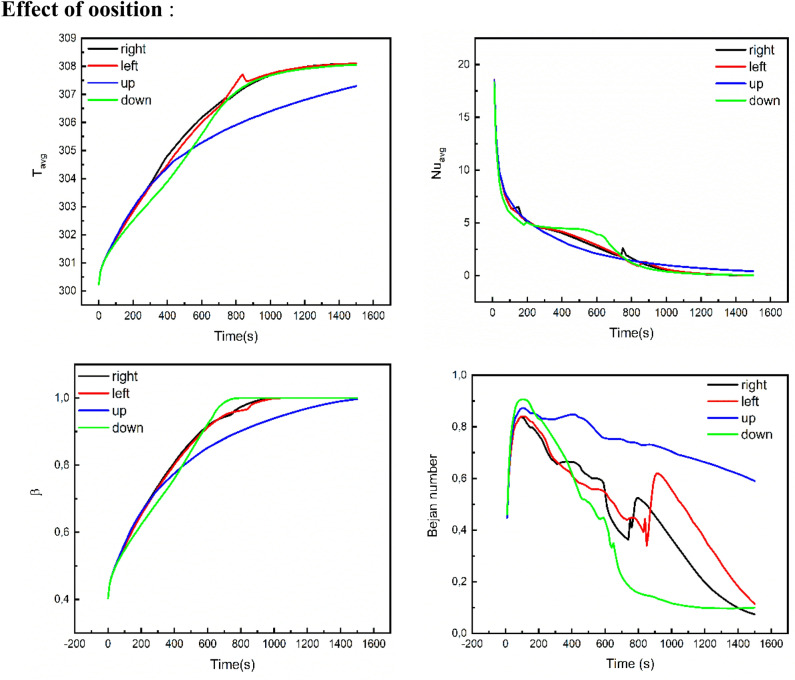
The finned tube position on the Average temperature, Average Nusselt number, Average liquid fraction (β) and Bejan number over time.

**Figure 9:**
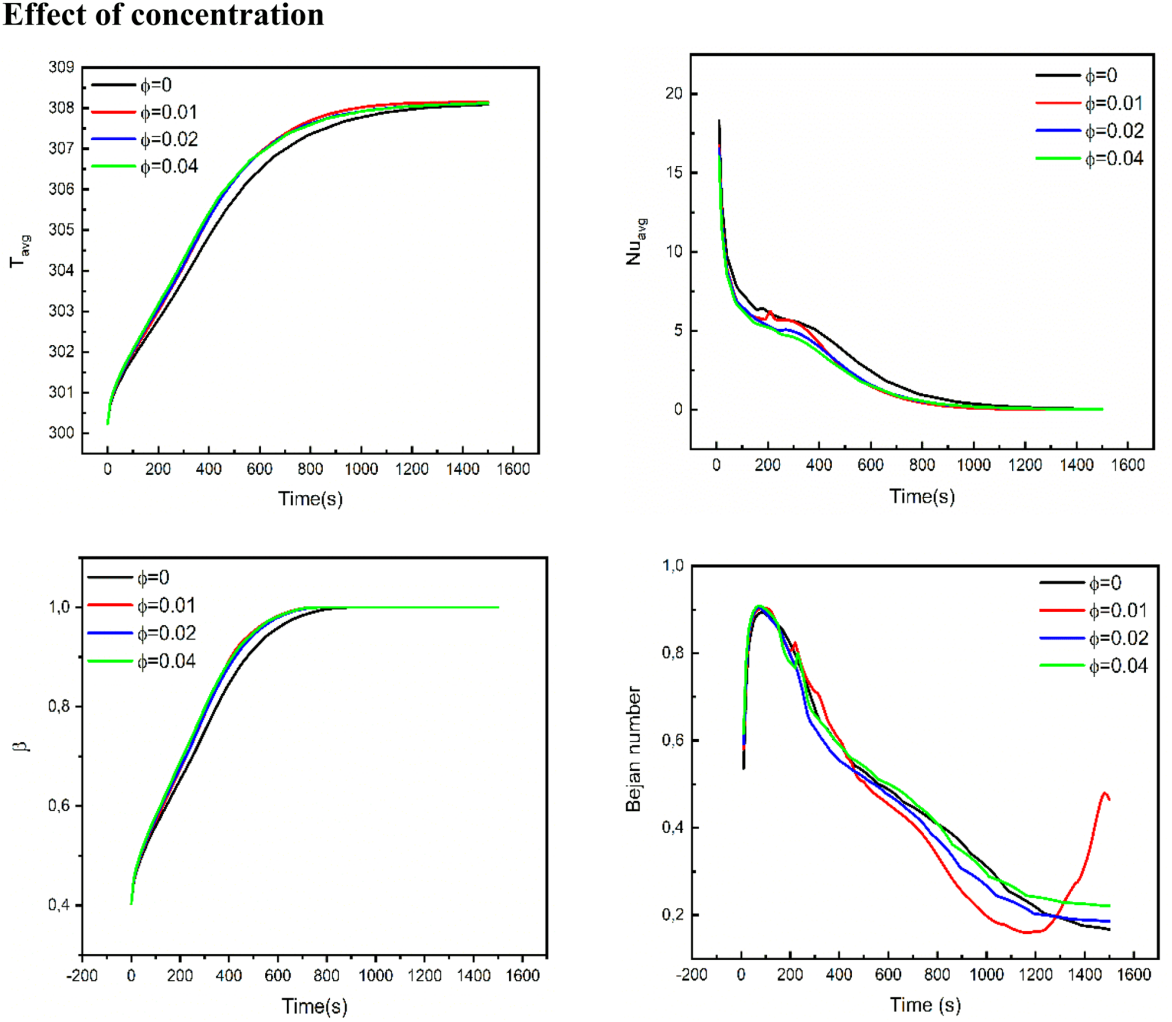
The influence of Np concentrations (∅) on the Average temperature, Average Nusselt number, Average liquid fraction (β) and Bejan number over time.

The influences of the rotation angle $\alpha$, location of the inner rotating shape, concentration of the NP and the considered range of the time on the average temperature ${T}_{avg}$, average Nu ${Nu}_{avg}$, melting rate $\beta$ and average ${Be}_{avg}$ are examined using Figs. 7, 8 and 9. ${T}_{avg}$ and melting rate $\beta$ obtained higher values in the case of the non-rotating shape while $\alpha =0$ gives low values of ${Be}_{avg}$ and ${Nu}_{avg}$. The figures also disclosed that when the rotating shape is placed in the upper part (Up-case) of the wavy domain, this causes a diminishing in ${T}_{avg}$, melting rate $\beta$ and temperature gradients, while the values of the average Be are reduced when the inner rotating shape is located in the lower part (down-case). Furthermore, the growth in $\phi$ leads to the enhancement of both of ${T}_{avg}$, melting rate $\beta$ and rate of HT due to the increase in the thermal edge layer near the boundaries.

## Conclusion

The melting process within a wavy circular cylinder, including rotating shapes, has been examined. The inner shape is a circular cylinder with three attached heated fins. The 2nd law of thermodynamics was applied to examine the irreversibility while the melting phenomena were simulated using the enthalpy-porosity approaches. The simulations started with mathematical formulations while the Galerkin FE technique was used to solve the governing system. Parametric studies were performed for the rotation angle, location of the inner rotating shape, concentration of NP and the considered range of the time. The following key findings are noted:

- Values of $\boldsymbol{\alpha }=0$ and $\boldsymbol{\alpha }={\varvec{\pi}}$ accelerate the mixture movements while $\boldsymbol{\alpha }=3{\varvec{\pi}}/2$ decrease the HT entropy at the lower wavy edge.
- Located the complex rotating shape in the down area of the flow domain gives the largest rate of the flow.
- Located, the rotating shape in the up regions causes a dominance of the HT entropy on most of the wavy domain.
- The melting process is augmented as the concentration of the NP is altered.
- As time progresses, the temperature gradients diminish while the opposite features are noted for the average temperature.
- ${T}_{avg}$ and melting rate $\beta$ obtaine higher values in case of the non-rotating shape while $\alpha =0$ gives a low values of ${Be}_{avg}$ and ${Nu}_{avg}$.
- The rate of HT, average temperature and melting rate are augmented as the NP concentration is growing.

## Data Availability

The numerical data used to support the findings of this study are included within the article.

## Abbreviations

g: Gravity
T_m_: Fusion temperature
C: Mushy zone constant
NEPCM: Nanoenhanced PCM
*L*_*f*_: Latent heat coefficient
k: Thermal conductivity
T_s_: Solidus temperature
FEM: Finite element method
*T*_I_: Liquidus temperature

## Greek symbols

*a*: Thermal diffusivity (m^2^/s)
*ρ*: Fluid density
*ϕ*: Nanoparticle volume fraction

## Subscripts

*nf*: NEPCM
*f*: Pure fluid
