# Author Correction: Melting enhancement of PCM in a finned tube latent heat thermal energy storage

**DOI:** 10.1038/s41598-022-21218-z

**Published:** 2022-09-28

**Authors:** Sameh Ahmed, Aissa Abderrahmane, Abdulkafi Mohammed Saeed, Kamel Guedri, Abed Mourad, Obai Younis, Thongchai Botmart, Nehad Ali Shah

**Affiliations:** 1 College of Science, King Khalid University, Abha, 61413 Saudi Arabia; 2 Department of Mathematics, Faculty of Science, South Valley University, Qena, 83523 Egypt; 3 Laboratoire de Physique Quantique de La Matière et Modélisation Mathématique (LPQ3M), University of Mascara, Algeria, Algeria; 4 Department of Mathematics, College of Science, Qassim University, Buraydah, Kingdom of Saudi Arabia; 5 Mechanical Engineering Department, College of Engineering and Islamic Architecture, Umm Al-Qura University, P.O. Box 5555, Makkah, 21955 Saudi Arabia; 6 College of Engineering at Wadi Addwaser, Mechanical Engineering Department, Prince Sattam Bin Abdulaziz University, WadiAddwaser, Saudi Arabia; 7 Department of Mathematics, Faculty of Science, Khon Kaen University, Khon Kaen, 40002 Thailand; 8 Department of Mechanical Engineering, Sejong University, Seoul, 05006 South Korea

Correction to: *Scientific Reports* 10.1038/s41598-022-15797-0, published online 07 July 2022

The original version of this Article contained an error in the spelling of the author Obai Younis, which was incorrectly given as Obai Younes.

Furthermore, the Acknowledgments section contained an error in the Grant Code,

“The authors extend their appreciation to the Deanship of Scientific Research at King Khalid University for funding this work through Larg Groups Project under Grant Number (R.G.P2/22/43). The authors would like to thank the Deanship of Scientific Research at Umm Al-Qura University for supporting this work by Grant Code: 22UQU4331317DSR09.”

now reads:

“The authors extend their appreciation to the Deanship of Scientific Research at King Khalid University for funding this work through Large Groups Project under Grant Number (R.G.P2/22/43). The authors would like to thank the Deanship of Scientific Research at Umm Al-Qura University for supporting this work by Grant Code: 22UQU4331317DSR31.”

The original Article has been corrected.

